# Distinct behavior of myelomonocytic cells and CD8 T cells underlies the hepatic response to *Listeria monocytogenes*

**DOI:** 10.12688/wellcomeopenres.12941.1

**Published:** 2018-04-24

**Authors:** Peter Velázquez, Cassandra Williams, Ingrid Leiner, Eric G. Pamer, Michael L. Dustin

**Affiliations:** 1 Molecular Pathogenesis Program, The Helen L. and Martin S. Kimmel Center for Biology and Medicine, Skirball Institute of Biomolecular Medicine, New York, NY, 10016, USA; 2 Department of Microbiology and Immunology, Indiana University School of Medicine – South Bend, South Bend, IN, 46617, USA; 3 Biotherapeutics, Thousand Oaks, CA, 91301, USA; 4 Atara Biotherapeutics, Westlake Village, CA, 91361, USA; 5 Infectious Disease Service, Department of Medicine, Memorial Sloan-Kettering Cancer Center, Immunology Program, Sloan-Kettering Institute, New York, NY, 10021, USA; 6 Kennedy Institute of Rheumatology, NDORMS, The University of Oxford, Oxford, OX3 7FY, UK

**Keywords:** T cell motility, T cell migration, TCR stop signal, Listeriosis, intravital microscopy, inflammation

## Abstract

**Background**: The immune response to *Listeria monocytogenes* (LM) is characterized by formation of leukocyte rich foci of infection in liver and spleen.  Although much has been gained in our understanding of immune response through the study of LM, little is known about spatio-temporal regulation of immune response to Listeria in liver.

**Methods:** We utilize a combination of molecular, genetic and intravital microscopic approaches to gain insight into the dynamics of foci and leukocyte behavior during hepatic Listeriosis.

**Results**: LM foci efficiently exclude blood flow, indicating the presence of a barrier separating the foci and healthy tissue.  Despite this barrier, sinusoidal myelomonocytic cells readily enter or transiently interact with cells at the edge of foci of infection.  Next, utilizing L9.6 transgenic CD8 ^+^ T cells specific for an endogenously processed LM antigen, p60 217-225, along with LM deficient in this epitope, we define the role of TCR in T cell migratory behavior in infected liver.  Surprisingly, T cell behavior varies with micro-anatomic locale.  Near foci, non-specific adhesion mechanisms dominate lymphocyte behavior.  Antigen specific effects on motility became detectable only distal to foci.

**Conclusions:** These data suggest that LM antigens act in a paracrine manner to mediate protection from Listeriosis in the liver.

## Introduction

*Listeria monocytogenes* (LM) is one of the best-studied pathogens in immunology and microbiology ( David & Cossart, 2017; Pamer, 2004; Portnoy *et al*., 2002). Insights into the pathophysiology underlying Listeriosis have significantly contributed to our understanding of the coordination of innate and adaptive immune responses and mechanisms of bacterial virulence. Several studies utilizing various pathogen models in combination with single and multi-photon intravital microscopic approaches have provided new insight into the leukocyte behavior underlying immune responses ( Aoshi *et al*., 2008; Chtanova *et al*., 2008; Egen *et al*., 2008; Waite *et al*., 2011). 

The temporal orchestration of the immune response to microbes in liver, as well as at other tissue sites, is well documented. Hepatic Kupffer cells capture LM and other sinusoidal microbes through Complement and scavenger receptors. This is followed by a rapid influx of neutrophils. Both of these processes play a role in early protection and lead to formation of infection foci ( Broadley *et al*., 2016; Surewaard & Kubes, 2017; Unanue, 1997; Witter *et al*., 2016) . In *Staphylococcus aureus* infection, release of Neutrophil Extracellular Traps (NETS) contribute to bacterial capture ( Kolaczkowska *et al*., 2015; McDonald *et al*., 2010). In hepatic Listeriosis, inflammatory monocyte influx proceeds soon after polymorphonuclear infiltration and are also critical for protection against LM ( Helmy *et al*., 2006; Shi *et al*., 2010). Neutrophils and monocytes can be visualized via intravital microscopy using mice, in which eGFP is targeted into the LysM locus ( Smiley *et al*., 1995); we will refer to the GFP ^+^ cells in these mice as myelomonocytic cells (MMC). CD8 T cells recognizing N-formylmethionine peptide in the context of H2-M3 respond 3–5 days post-infection in mice, although their role in human Listeriosis remains unclear ( Cho *et al*., 2011; Gulden *et al*., 1996). Importantly, conventional CD4+ and CD8+ T cells migrate into the liver as early as day 3 post infection and this response peaks during days 5–7 ( Lenz *et al*., 1996; Unanue, 1997). Each CD8+ or CD4+ T cell responses are sufficient in providing sterilizing immunity and dissolution of foci of infection ( Lara-Tejero & Pamer, 2004). While the cell types and kinetics of immunity to LM is well documented, little is known about the leukocyte behavior in response to LM at effector sites.

Previous studies have identified Listeria-derived immunodominant peptide epitopes presented on H2-Kd and have characterized the resultant T cell repertoire ( Harty & Pamer, 1995; Pamer, 1994; Sijts *et al*., 1996; Vijh & Pamer, 1997; Vijh *et al*., 1998). One naturally occurring dominant peptide epitope is derived from p60 protein, peptide 217–255 ( Vijh & Pamer, 1997). Polyclonal and monoclonal T cells generated from infected mice that are specific for this epitope provide protective immunity against Listeriosis ( Harty & Pamer, 1995); CD8 ^+^ L9.6 transgenic T cell receptor, specific for p60 217–225, presented in the context of H2-Kd, was cloned from monoclonal T cells protective against LM ( Harty & Pamer, 1995).

In previous studies, we engineered LM expressing p60 antigen with a single point mutation in the MHC anchor residue 218, changing it from a tyrosine to a serine, termed 218S ( Harty & Pamer, 1995). This diminished 217–225 epitope generation by LM infected cells to below the limit of detection. *In vivo*, 218S did not elicit T cells specific for 217–225, while T cell proliferation, target cell lysis and cytokine production against other epitopes remained intact. Also, LM virulence in spleen remains unchanged between wild type and 218S ( Harty & Pamer, 1995; Vijh *et al*., 1998). Therefore, 218S mutants do not present p60 217–225 peptide antigen *in vivo*.

Here, we utilize multi-photon intravital microscopy to gain insight into the orchestration of the leukocyte response to LM *in vivo*. LM foci are characterized by reorganization of the hepatic sinusoids, mediated by MMC, leading to the exclusion of blood perfusion. MMC migration is characterized by entry but not exit from foci. Additionally, some MMC migrate tangentially to the perimeter of the foci. Next, we report that TCR specificity does not play a role in the patrolling behavior of LM specific CD8 ^+^ T cells proximal to foci. Conversely, distal to foci of infection CD8 ^+^ T cell antigen specificity does play an important role in defining patrolling behavior. We define the relationship between TCR specificity and T cell patrolling and, discuss the mechanistic importance of these observations in protection from hepatic Listeriosis.

## Methods

### Listeria monocytogenes infection

LM challenge doses were optimized for each strain of mice, either C57BL/6 or CB6F1 (C57BL/6 x Balb/c), male or female animals 8–12 weeks of age and weighted approximately 25–30g at the time of challenge. Animals were housed in ventilated cages, up to four animals per cage with bedding changed twice weekly. Optimal challenge doses were determined by the ability to grossly identify Listeria foci at day 3 and 5 post-infection. Challenge doses with LM expressing red fluorescent protein (RFP) or 218S as high as 2.5 × 10 ^4^ led to a lethality of less than 10% at day 5 post-infection. RFP is a targeted gene insertion into the endogenous actA1 locus and expression is driven by the endogenous promoter. Similarly, 218S is a targeted mutation to the endogenous p60 gene and expression is driven by the endogenous locus. RFP Listeria were a generous gift from Dr. Dan Portnoy. 218S Listeria were previously reported (Vijh and Pamer 1998). Work with mice was performed in an ethical manner under supervision of the NYU School of Medicine Institutional Animal Care and Use Committee (protocol 090412-03).

### Surgery and anesthesia

Animals were anesthetized by intra-peritoneal injection of a solution (~100 µl) delivering ketamine (50 mg/Kg), xylazine (10 mg/Kg), and acepromazine (1.7 mg/Kg). Anesthesia was maintained during image acquisition with one-half dose s.c. boosting every 45 min for up to 2.5 h. For surgery, the anesthetized animal was placed on a heating pad set at 37°C. Left-side abdominal fur was trimmed, and the liver was exposed through a 1.5-cm horizontal incision. The hepatoform ligament was then cut and the tip of the left lobe of the liver gently exposed.

### Intravital imaging

All surgical procedures are approved by NYU- School of Medicine IACUC. Images were acquired with Zeiss Plan-Apochromat 20X/0.75 objective on ZEISS LSM 510. Intravital imaging was conducted as previously described ( Velázquez *et al*., 2008). In brief, upon completion of the surgery, animals were placed with the abdominal side down on a custom-made stage insert (Ludl Electronic Produces, Hawthorne, NY, USA), in which a coverslip (No. 1.5: 0.16–0.19 mm thick) was mounted near the center and narrow strips of paper (1.5 mm × 1.5 cm) was glued. These strips of paper provided friction that help immobilize the tissue for imaging.

The stage insert was then placed on an inverted microscope stage and imaged. For time-lapse image acquisition, 1 volume was collected every 30 s, with z-slices acquired every 5 μm. The maximum depth acquired over a 30-s interval with a single-photon light source was 20 μm utilizing a 2 μs dwell time. The animal is maintained at 37°C in an environmental chamber with supplemental medical-grade oxygen supplied via a nose cone. To visualize blood perfusion, mice were treated with an intravenous dose of low molecular weight 10 kDa dextran conjugated to Alexa-647 fluor (Molecular Probes) while imaging. Animals were euthanized at the end of each experiment.

### Leukocyte migration analysis

*In vivo* hepatic leukocyte migration was quantified using Volocity Version 4.3 (Improvision Inc.). Each cell was tracked semi-automatically with the centroid gated on size and intensity and, confirmed visually during each frame collected. For analysis of each time-lapse image, every cell is examined during each acquisition frame (1/30 seconds). Volocity data analysis output included speed, displacement, maximum displacement and track length. Arrest coefficient is calculated as the percent of time a cell migrates less than 2 microns per minute. Meandering index is displacement as a function of track length. Confinement index is the maximum displacement as a function of track length.

### L9.6 *in vitro* stimulation

L9.6 CD8 T cell transgenic animals were crossed onto Rag null animal expressing EGFP transgene driven under the actin promoter. For transfer studies, L9.6 T cells were sorted via MACS (Miltenyi Inc) mouse untouched CD8 T cell isolation kit. 5 × 10 ^5^ L9.6 T cells were stimulated *in vitro* on 5 × 10 ^7^ irradiated (2000 rad) splenocytes, with 10 nM peptide in the presence of 25 units/ml IL-2. After 4 days, cells were expanded in fresh media with 25 units/ml IL-2 in the absence of APCs and peptide to allow for rest. Two days following expansion, T cells were transferred into syngeneic recipients for imaging.

### Adoptive transfer

At day 3 post infection, 5 × 10 ^6^ L9.6 T cells (day 2 post-expansion) were transferred into recipient animals. At 4–6 hours post-transfer, animals were anesthetized and prepared for imaging via single- or multi- photon intravital microscopy.

### Statistics

Statistical analysis was conducted using GraphPad Prism 5 software. All data sets were examined for Gaussian distribution via a D’Agostino and Pearson normality test. For determination of significance of differences between two groups, a two-tailed nonparametric Mann-Whitney test (non-Gaussian) with a 95% confidence interval was conducted. Significance is defined as p ≤ 0.05. 

## Results

### Ischemic perfusion in LM foci of infection

**Figure 1.**
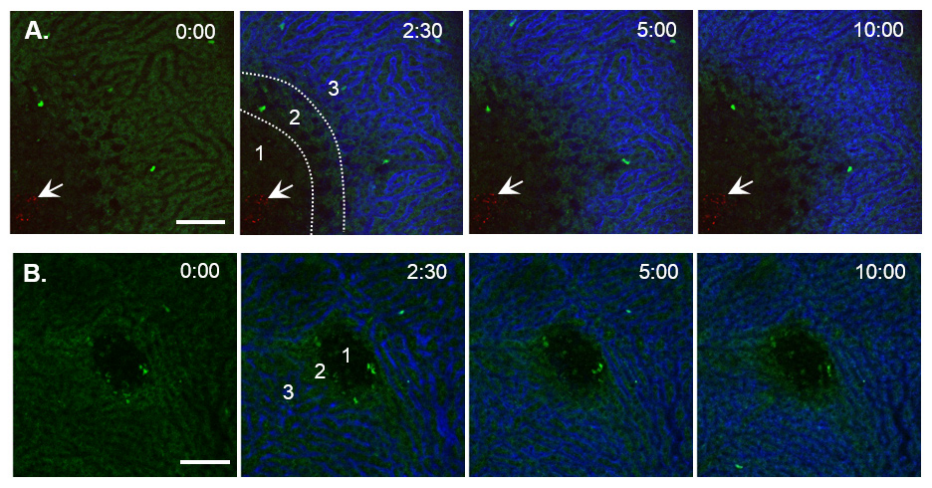
Lack of perfusion of LM foci. **A** and **B**, CB6F1 animals were imaged at day 3 post-infection with 2 x 10 ^4^ LM-RFP (red) and eGFP-L9.6 transgenic T cells (green). Mice were injected with 10 kDa Alexa-647 (blue) i.v. while imaging. Three distinct regions are identifiable; foci proper (1), foci border (2) and perfused normal liver parenchyma (3). **A**, large foci with LM-RFP. **B**, small foci lacking visible LM-RFP is likely the top of a deeper focus. Scale Bar = 100 μm.

LM infection is, in part, characterized by leukocyte filled foci. To better understand the microanatomy of this structure, we challenged CB6F1 mice with LM expressing red fluorescent protein (LM-RFP) and visualized LM foci in live mice at day 3 post-infection at the surface of the liver by intravital microscopy. To visualize blood perfusion, CB6F1 mice were treated with an intravenous dose of low molecular weight 10 kDa dextran conjugated to Alexa-647 fluor (Molecular Probes) while imaging. Hepatic auto-fluorescence excited by 488 nm and measured at 530 nm, was used to delineate metabolically active liver. We defined 3 regions in relation of the foci. Exclusion of the fluorescent dextran, lack of auto-fluorescence, and presence of LM-RFP characterized region 1 ( Figure 1A, Supplemental Video 1). Region 2 was a transitional zone of ~100 μm, in which blood perfusion was absent, but hepatocyte autofluorescence was detected ( Figure 1A and B). Region 3 had blood perfusion and autofluorescence characteristics similar to healthy liver ( Figure 1A and B). We never detected LM-RFP outside of region 1. When the center of the foci are near to the capsule, the foci appear >150 μm in diameter and display the characteristics above. We interpret small foci (<150 μm) as having deeper centers that may represent region 2 with variable autofluoresence and perfusion ( Supplemental Video 2) and these were scored based on foci diameter measured in Volocity software ( Table 1). 

**Table 1.** Size of foci of infection.

	Listeria		
**Size (µm)**	**WT**	**218S**	**Total**
**0-150**	1/1	1/1	2/2
**150-300**	0/7	1/5	1/12
**>300**	0/6	0/1	0/7

# perfused / total # of foci in viewing field. WT n=12 mice; 218S n=4 mice

### MMC behavior in LM foci

**Figure 2.**
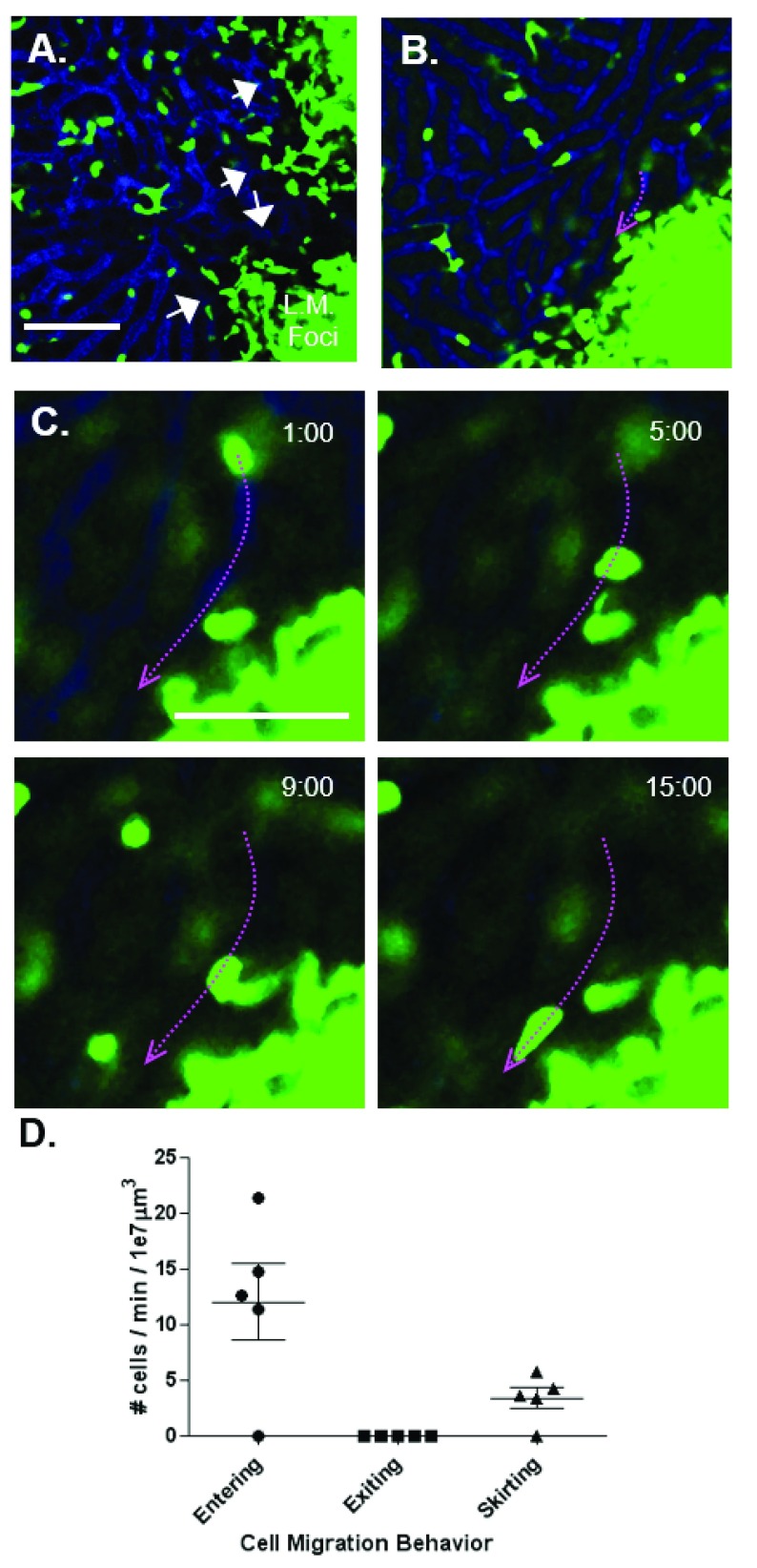
MMC behavior near LM foci. **A**– **C**, MMCs (green) in LM infected liver injected intravenously with 10 kDa dextran Alexa-647 (blue). **A**, Distinct entry points into foci from the sinusoid (arrows). **B**, Track (lavender arrow) of a MMC ‘skirting’ foci. **C**, Time-lapse of ‘skirting’ behavior. **D**, Quantification of MMC migratory behavi0or. **A**– **B**, scale bar = 100 mm. **C**, scale bar = 50 mm. n=4.

MMCs play a critical role in early protection from LM and are critical contributors to formation of foci. To better understand the contribution of MMCs to exclusion of blood perfusion, *LysM ^+/EGFP^* reporter animals were challenged with LM-RFP and examined at day 3 post-infection. As expected, LM foci were filled with MMCs, which disrupted the sinusoidal structure and excluded 10 kDa dextran ( Figure 2). We were particularly interested in examining if cells were capable of migrating in and out of foci. Image analysis revealed two distinct phenotypes: sinusoidal MMCs that entered foci ( Figure 2A and Supplemental Video 3); or that did not enter foci but interacted with MMCs inside the foci proper as they migrated tangentially to the sinusoid-foci border ( Figure 2B and C, Supplemental Video 4). This later behavior is characterized as ‘skirting.’ Quantitative analysis showed that an average of 12 ± 3.47 cells per minute per 1 × 10 ^7^ μm ^3^ entered foci while no cells were observed migrating out. 1 × 10 ^7^ μm ^3^ is an approximate volume of a single focus of infection assuming a ~150 μm radius and spherical shape. MMCs ‘skirt’ foci at a rate of 3.14 ± 0.94 cells per minute per 1 × 10 ^7^ μm ^3^ ( Figure 2D). Therefore, foci are accessible to migrating MMCs found in liver sinusoids. 

### Antigen specific CD8 T cell behavior in Listeriosis

**Figure 3.**
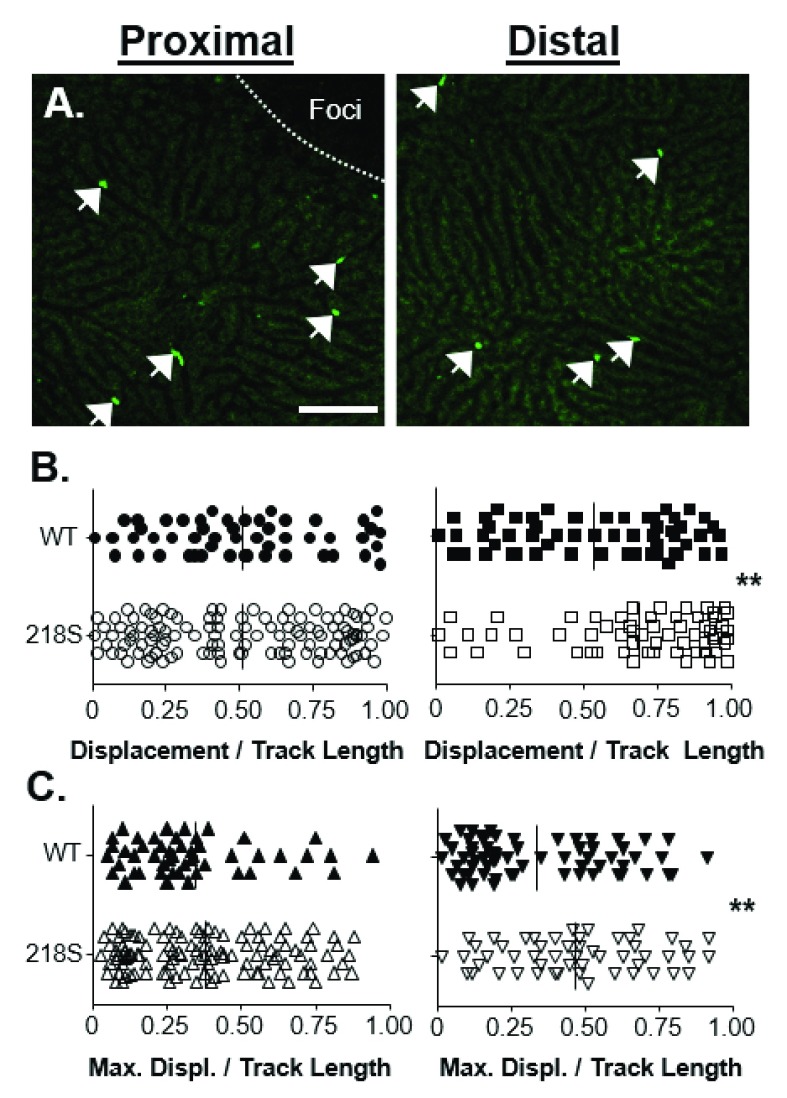
Persistence of T cell migration in liver parenchyma. **A**– **C**, *In vitro* primed CD8+ L9.6 transgenic T cells (arrowheads) were transferred into antigen positive LM-WT or antigen negative LM-218S infected CB6F1 animals at day 3 post-infection. Intravital images were acquired proximal (left) and distal (right) to LM foci. No significant difference in Meandering Index ( **B**, displacement / track length) or Confinement Index ( **C**, maximum displacement / track length) were observed proximal to foci of infection; p=0.97 and p=0.43, respectively. Conversely, distal to foci L9.6 T cell migration was more linear ( **B**, right) and less confined ( **C**, right); p=0.004 and p<0.004, respectively, with LM-218S compared to LM-WT infection. n = 4 mice. Each data point represents an individual cell.

CD8 T cells are key contributors in protection against LM and in providing sterilizing immunity. Therefore, to better understand the role antigen specificity plays in liver CD8 T cell migration during Listeriosis, L9.6 transgenic T cells were utilized. To study the role of antigen specificity, *in vitro* primed L9.6 cells were transferred into recipient animals infected with either antigen positive LM-RFP (wild type) or antigen negative LM-218S and were visualized at day 3 post infection. There were no differences in the foci formed by WT or 218S LM ( Table 1), consistent with normal virulence of LM-218S ( Vijh *et al*., 1998). T cells were never observed in region 1 and rarely entered region 2 ( Table 2). We extended our analysis of region 3 to areas within the same field with the focus, within 400 µm ( Figure 3A, left), as well as areas distal to the focus, greater than 400 µm away ( Figure 3A, right). First, the directed motion of L9.6 T cell migration was examined by quantifying meandering index (displacement/track length) and confinement index (maximum displacement/track length) ( Figures 3B and C, respectively). Proximal to foci, no role for antigen specificity was observed in the meandering (p=0.97) or confinement (p=0.38) of T cell migration ( Figure 3B left and Figure 3C left, respectively). In contrast, distal to foci, we observed an increase in the meandering index (p=0.004), and a larger confinement index (p=0.004) in the absence of antigen compared to the presence of antigen ( Figure 3C, right). This data indicates that T cell movement is restricted by innate factors proximal to foci and that the influence of antigen is only detected greater than 400 μm from the foci, which was not expected. These results suggest that L9.6 cells detect LM antigens over “paracrine” distances in the liver.

**Table 2.** Frequency of T cells near foci of infection

Listeria	T cell f	n (mice)	p
WT	13±7	4	p=0.68
218S	17±11	4	

**Figure 4.**
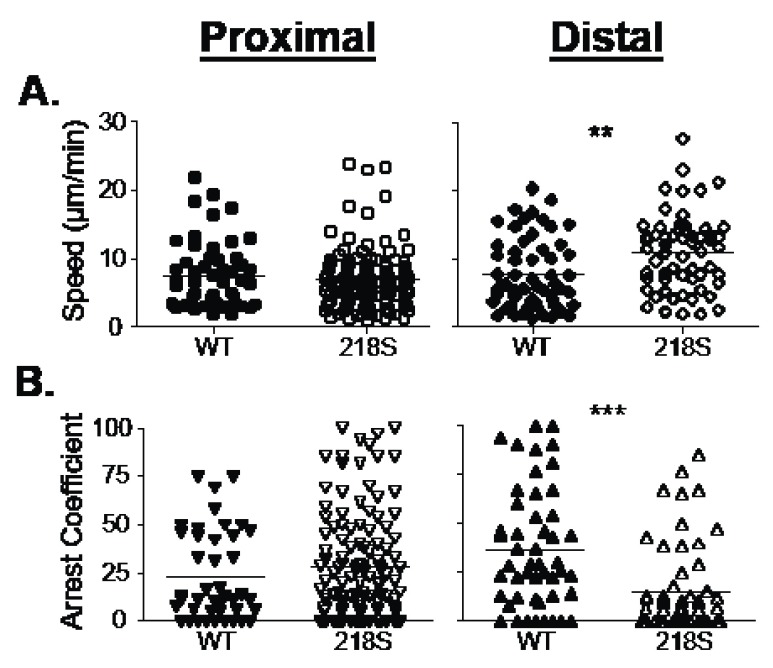
Speed and arrest of T cell migration in liver parenchyma. **A**– **B**, *In vitro* primed CD8 ^+^ L9.6 transgenic T cells were transferred into LM infected CB6F1 animals as in Figure 3. Intravital images were acquired proximal (left) and distal (right) to LM foci. No significant difference in T cell speed ( **A**) or arrest coefficient ( **B**) were observed proximal to foci; p=0.60 and p=0.32, respectively. Conversely, in the absence of cognate antigen (218S) L9.6 T cells migrated faster ( **A**, right) and arrested less ( **B**, right); p=0.002 and p<0.0001, respectively. n = 4 mice. Each data point represents an individual cell.

To further characterize the role of antigen specificity in T cell migration, speed and arrest coefficient were analyzed. Proximal to foci, there was not a significant difference in speed (p=0.60) or arrest coefficient (p=0.32) without or with antigen ( Figure 4A and B, left). In contrast, distal to foci antigen slowed migration (p=0.002) and increased the arrest coefficient (p<0.0001), compared to 218S antigen null foci ( Figure 4A and B, right). These results further support the notion that antigen is recognized greater than 400 μm from the focus.

## Discussion

Much of our understanding of the biology and kinetics of the cell-mediated immunity against intracellular microbes comes from studies on Listeriosis. Here, we provide a dynamic view of the cell behavior that underlies immunity to this microbe in the liver. We describe the lack of blood perfusion in foci of infection and observe the dynamic behavior of MMCs as they interact with foci. Contrary to our expectations, effector CD8 T cells did not penetrate into and directly interact with infected cells in foci as has been observed for CD4 T cells in mycobacteria induced liver granuloma ( Egen *et al*., 2008). Instead, the effector CD8 ^+^ T cells detected LM antigen p60 hundreds of microns away from the live LM in the foci, suggesting paracrine communication between foci and surrounding liver tissue. While TCR specificity is critical for T cell reactivity, as is well known, TCR specificity does not play a role in patrolling behavior near foci of infection. Rather non-specific mechanisms, likely integrin mediated, dominate migratory behavior near foci. Distal to foci, non-antigen specific T cells migrate faster than antigen specific T cells, allowing increased scanning of antigen presenting cells.

We divided the infected liver into 3 regions. Region 1 contained live LM and lacked substantial perfusion. This region also had depressed hepatocyte metabolism based on lack of flavoprotein auto-fluorescence, which is reminiscent of ischemic liver tissue ( Geissmann *et al*., 2005; Scholz *et al*., 1969). Region 2 is a transitional zone in which blood flow is absent or intermittent, but hepatocyte metabolism is evident. This zone is ICAM-1 and hyaluronic acid rich and corresponds to an entry pathway for CCR2 ^+^ inflammatory monocytes ( Shi *et al*., 2010). Models of sterile liver injury display a similar architecture in which platelets, fibrin and neutrophils deposit in a barrier zone that can be traversed by MMC, but not by T cells ( Jorch & Kubes, 2017; McDonald *et al*., 2010). Consistent with an important role for such a barrier, mice that lack MMCs or fibrinogen do not form foci and succumb to overwhelming infection ( Mullarky *et al*., 2005; Unanue, 1997). Region 3 had well perfused sinusoids and metabolically active hepatocytes. Proximal regions display a gradient of ICAM-1 and hyaluronic acid expression radiating out from region 2. Distal regions appear healthy, but p60 antigen derived peptides were presented in region 2 and 3 at sufficient density to induce antigen specific T cell confinement and arrest. The p60 antigen is secreted into the cytoplasm of infected cells and is very efficiently processed with 1 L ^d^-p60 217-225 complex generated for every 15 p60 molecules degraded ( McDonald *et al*., 2010). How this paracrine communication of antigen through the tissue is mediated is an intriguing question.

Although ischemic foci are accessible to migrating MMCs found in liver sinusoids, MMC may enter foci to provide direct anti-LM activity. We hypothesize that cells ‘skirting’ the foci may capture antigen through direct cell-cell interaction with MMCs in the foci proper and carry that antigen to a distal location or Kupffer cell. This latter mechanism may provide a mode to distributing antigen to Kupffer cells distal from foci, thereby increasing the probability of LM specific T cells engaging cognate antigen. Another possible mechanisms for dissemination of peptides in the liver is through gap junctions that interconnect hepatocytes and Kupffer cells ( Eugenín *et al*., 2007). Gap junctions relay antigenic peptides between dendritic cells ( Neijssen *et al*., 2005).

During the course of hepatic Listeriosis, endogenously primed T cells begin to arrive at foci at day 3 post-infection. In this study, we aimed to mimic early events in T cell-LM foci interactions. Therefore, we examined effector T migration into foci at day 3 post-infection. At later time points, days 5, 6 and 7, the variability in the foci presence and/or anatomic microanatomic locale prohibited visualization. In this study, we utilized an adoptive transfer system of *in vitro* primed CD8 T cells to understand migration near or distal to foci. In establishing this system, we titrated the number of donor cells to minimize effects of the transfer system on cell migration. It is important to note that studies have documented variability in T cell priming and memory formation dependent on donor cell numbers. Nonetheless, when controlling donor cell numbers, we report a dependence of TCR specificity on cell patrolling.

 Taken together, we propose a model where early formation of foci, which plays an important role in protection from sepsis, is driven in part by formation of a barrier to blood perfusion that is passable by MMCs. During the course of infection, some bacteria may escape foci and seed new foci of infection. By day 3 post infection, primed CD8 effector T cells arrive in the liver where they migrate in perfused sinusoids, scan APCs in an antigen independent manner and undergo antigen dependent activation and arrest. Paracrine communication of antigen from the foci to hepatic sinusoids activates CD8 T cells that may in turn enhance Kupffer cells and MMC listeriacidal activity through release of cytokines such as IFN-γ ( Pamer, 2004). The enhanced listeriacidal activity may then contribute to destruction of live LM in foci and protect from formation of new foci. In this model, CD8 T cells do not need to directly interact with an infected cell in foci, but appear to operate through an unexpected antigen specific response to the infected organ. 

## Data availability

Raw data for this study are available on OSF http://dx.doi.org/10.17605/OSF.IO/TR3EV ( Dustin, 2018)

Data are available under the terms of the Creative Commons Zero "No rights reserved" data waiver (CC0 1.0 Public domain dedication).

## Supplementary material

**Supplementary File 1: Supplementary videos.**

**Supplemental Video 1: Time-lapse of Listeria infected liver post transfer of L9.6 CD8 T cells.** Part of a large (>150 µm) focus of infection visualized while injecting 10 kDa Alexa 647 i.v. (blue). Viewing field is 450 × 450 µm. RFP-Listeria (red) are visible at the center of the focus. L9.6 T cells (bright green) navigate the surrounding healthy tissue and edge of the focus only.
**Supplemental Video 2: Time-lapse of Listeria infected liver post transfer of L9.6 CD8 T cells.** A small (<150 µm) focus of infection visualized while injecting 10 kDa Alexa 647 i.v. (blue). Viewing field is 450 × 450 µm. RFP-listeria are not visible, but the L9.6 T cells (bright green) navigate the surrounding healthy tissue and the edge of the focus.
**Supplemental Video 3: Time-lapse of LysM ^EGFP/+^ liver 3 days post-infection with LM.** Two large foci filled with myelomonocytes (bright green), visualized while injecting 10kDa Alexa 647 i.v. (blue). Cell migration tracks highlight myelomonocytes that move from the sinusoid into the foci. Viewing field is 350 × 350 µm.
**Supplemental Video 4: Time-lapse of LysM ^EGFP/+^ liver 3 days post-infection with LM.** Viewing field is focused on a single large foci filled with myelomonocytes (bright green), visualized while injecting 10 kDa Alexa 647 i.v. (blue). Cell migration tracks highlight myelomonocytes ‘skirting’ the foci. Viewing field is 350 × 350 µm.
