# Supplementary material for: Distinct behavior of myelomonocytic cells and CD8 T cells underlies the hepatic response to Listeria monocytogenes
Source: Wellcome Open Res. 2018 Apr 24;3:48. [Version 1] doi: 10.12688/wellcomeopenres.12941.1 (PMC5958313; doi:10.12688/wellcomeopenres.12941.1)
Supplement: Supplementary file 1 [file wellcomeopenres-3-14032-s0000.tgz › 6a4077b8-1c6a-416c-a384-fa3bb2cd12dc.zip!/Supplementary File 1/Supplemental Video Legends.doc]

Supplemental Video I

Time-lapse of Listeria (red) infected liver post transfer of L9.6 CD8 T cells. Viewing field is focused on a large foci of infection visualized while injecting 10kDa Alexa 647 i.v.. Viewing field is 450 x 450 µm.

Supplemental Video II

Time-lapse of Listeria infected liver post transfer of L9.6 CD8 T cells. Viewing field is focused on a large foci of infection visualized while injecting 10kDa Alexa 647 i.v.. Viewing field is 450 x 450 µm.

Supplemental Video III

Time-lapse of LysM<sup>EGFP/+</sup>liver 3 days post-infection with LM. Viewing field is focused on two large foci filled with myelomonocytes, visualized while injecting 10kDa Alexa 647 i.v.. Cell migration tracks highlight cells that migrate from the sinusoid into the foci. Viewing field is 350 x 350 µm.

Supplemental Video IV

Time-lapse of LysM<sup>EGFP/+</sup>liver 3 days post-infection with LM. Viewing field is focused on a single large foci filled with myelomonocytes, visualized while injecting 10kDa Alexa 647 i.v.. Cell migration tracks highlight cells ‘skirting’ the foci. Viewing field is 350 x 350 µm.
