# Supplementary material for: Distinct behavior of myelomonocytic cells and CD8 T cells underlies the hepatic response to Listeria monocytogenes
Source: Wellcome Open Res. 2018 Apr 24;3:48. [Version 1] doi: 10.12688/wellcomeopenres.12941.1 (PMC5958313; doi:10.12688/wellcomeopenres.12941.1)
Supplement: Peer review history

# Peer review: Referee response for version 1

**DOI:** 10.21956/wellcomeopenres.14032.r33027

**Published:** 2018-05-17

**Authors:** Paul Kubes

**Affiliations:** 1 Department of Physiology and Pharmacology, Cumming School of Medicine, Calvin, Phoebe and Joan Snyder Institute for Chronic Diseases, University of Calgary, Calgary, AB, T2N 4Z6, Canada

The authors report the behaviour of CD8 T cells and monocytes/nneutrophils in Listeria infections.  I have a few points to make.

1. Are the Kupffer cells alive in area 1, 2 or 3?  If the CD8 T cells are interacting with Kupffer cells it would be interesting to see where the Kupffer cells are.  They may be dead in area 1.
2. What is evidence that CD8 T cells are critical for the LM response.  It would be good to better justify the reason to study CD8 T cells. 

IT would also be interesting to know what the CD8 T cells do later in the infection.  At day 3 they are only just arriving.

I have read this submission. I believe that I have an appropriate level of expertise to confirm that it is of an acceptable scientific standard.

# Peer review: Referee response for version 1

**DOI:** 10.21956/wellcomeopenres.14032.r33025

**Published:** 2018-05-14

**Authors:** Matteo Iannacone

**Affiliations:** 1 Division of Immunology, Transplantation and Infectious Diseases, Experimental Imaging Center , IRCCS San Raffaele Scientific Institute, Vita-Salute San Raffaele University, Milan, Italy

Velazquez and coworkers investigated the behavior of myelomonocytic and CD8 T cells within the liver upon Listeria monocytogenes infection. The Authors found that Listeria foci exclude blood flow, yet allow myelomonocytic cells to enter the foci or to transiently interact with cells at the edge of the foci. Such “skirting” is proposed to carry antigen to distal locations. Accordingly, CD8 T cells do not recognize antigen within the foci but do so hundreds of microns away.

 Overall, the work is interesting, well performed and the conclusions are supported by the data. The study is discussed in the context of prior literature, particularly the work from the Germain group on mycobacteria.

 A few suggestions might be worth considering:

1. The Authors interpret small foci (<150 um) as having deeper centers. Might they be foci where Listeria has been already cleared?
2. The size and brightness of GFP+ cells in LysM+/EGFP reporter mice has been used in the past to distinguish between neutrophils and monocytes/macrophages. Are the Authors able to do this in their experimental setup? If so, do neutrophils and monocytes/macrophages exhibit distinct behaviors upon Listeria infection?
3. Does the size of infection foci influence myelomonocytic cell behavior?
4. Can the Authors detect antigen-recognizing CD8 T cells in tissue sections (perhaps by virtue of IFN-g staining)? Are they indeed localized far away from the infection foci?
5. The co-transfer of an effector CD8 T cell population that is specific for an irrelevant antigen would strengthen the conclusion on the role of antigen in T cell migration upon Listeria infection.

I have read this submission. I believe that I have an appropriate level of expertise to confirm that it is of an acceptable scientific standard.
